# How to Adequately Report Workplace Violence in Healthcare Setting: A Systematic Review With Hierarchical Cluster Analysis of Workplace Violence Reporting Forms

**DOI:** 10.1155/jonm/4803748

**Published:** 2026-06-28

**Authors:** Sheng Qian Yew, Mohd Syazmin Zuwairy Mohd Sharial, Hanizah Mohd Yusoff

**Affiliations:** ^1^ Department of Public Health Medicine, Faculty of Medicine, Universiti Kebangsaan Malaysia, Jalan Yaacob Latif Bandar Tun Razak Cheras, Kuala Lumpur, 56000, Malaysia, ukm.my

**Keywords:** aggression, doctors, hospital, nurses, reporting

## Abstract

**Introduction:**

This review aimed to identify existing workplace violence (WPV) reporting forms worldwide, examine their domains and determine the essential information required to develop a more comprehensive and effective WPV reporting form.

**Materials and Methods:**

A systematic search of PubMed, Web of Science and Google Scholar from 1990 to 2025 was conducted. Data extracted included study characteristics, populations, reporting form names, number of items and reporting approaches. Descriptive analysis and hierarchical cluster analysis were performed to determine the number of items and domains included in each WPV reporting form.

**Results:**

A total of 22 WPV reporting forms were included. Across these forms, 148 reporting items were identified and grouped into 13 domains: sociodemographic data of the victim, job characteristics of the victim, characteristics of the notifier, characteristics of the incident, characteristics of the injury, measures taken during the violence, consequences of the violence, postviolence treatment, aftermath of the violence, reporting of the violence, perception of victim postviolence, characteristics of the assailant and characteristics of the witness. Hierarchical cluster analysis classified the 22 WPV reporting forms into three clusters based on comprehensiveness: rapid forms (three items), brief forms (mean 13.75 items) and detailed forms (mean 27.77 items).

**Conclusions:**

Substantial variation exists in the content of WPV reporting forms. Future WPV reporting forms should, at minimum, include seven most commonly reported domains, particularly job characteristics of the victim, characteristics of the incident, characteristics of the injury, measures taken during the violence, characteristics of assailant, characteristics of the witness and reporting of the violence.

**Implications for Nursing Management:**

The findings highlight substantial variability in the content, structure and domains of existing WPV reporting forms used in healthcare institutions, indicating the need for standardised, evidence‐based reporting tools that capture essential information relevant to nursing practice. Standardisation would improve comparability of WPV data across institutions and countries, facilitating more accurate benchmarking and global surveillance, particularly as nurses constitute the largest proportion of frontline healthcare workers. Additionally, nursing leaders, hospital administrators and health authorities can utilise these findings to strengthen institutional WPV policies by implementing clear and accessible reporting procedures and integrating domains related to prevention programmes, incident management and postincident support to guide policy development, staff training and resource allocation.

## 1. Introduction

Workplace violence (WPV) is recognised by the International Labour Office (ILO) as one of the most serious occupational hazards [1]. The Occupational Safety and Health Administration (OSHA) defines WPV as any act or threat of physical violence, harassment, intimidation or other threatening disruptive behaviour that occurs at the workplace [2]. In general, WPV can be categorised into four main types based on the assailant involved, namely Type I (criminal intent), where the assailant has no legitimate relationship with the workplace, Type II (patient/visitor), involving clients, patients or customers, Type III (worker‐on‐worker), involving violence among employees [3] and Type IV (organisational), where the violence arises from organisational practices or structures [4]. However, the current review focuses specifically on Type II WPV, which can be further classified into physical assault, verbal abuse, racial discrimination and sexual harassment [5].

It is noteworthy that nurses experience a higher risk of WPV than those in other industries [6]. Factors contributing to this include overcrowded emergency departments with large patient volumes [7], long waiting times for medical care and excessive hospital bureaucracy, all of which can lead to patient dissatisfaction [8, 9]. This dissatisfaction often manifests as violent behaviour directed at nurses by patients, their families or companions. Additionally, factors such as disease‐related stress, patient pain [10] and poor quality of healthcare services [6] further heighten the risk of WPV against nurses. Globally, WPV in healthcare facilities is reportedly high in prevalence and keeps increasing [11]. A systematic review [12] consisted of 136 articles reported that the overall prevalence of WPV ranged between 25.0% and 66.9%. Specifically, verbal violence is the most prevalent type of WPV, followed by physical violence [7].

WPV has significant consequences at individual, organisational and societal levels. Individually, nurses may suffer physical injuries and mental disturbance, such as posttraumatic stress disorder, anxiety, depression and physiological symptoms such as chest tightness and headaches, alongside reduced self‐confidence, job satisfaction and motivation [13–19]. Gender and sexual harassment further hinder career advancement, exacerbate inequities and reduce institutional diversity, ultimately affecting patient care [20]. Organisationally, WPV results in economic losses due to sick leave, compensation claims and staff turnover, with hospitals reporting costs up to USD 94,156 for injured staff and nationwide annual losses estimated at USD 4.2 billion [15, 21]. It also weakens teamwork, decreases efficiency and leads physicians to avoid high‐risk procedures, thereby lowering the quality of care [22, 23]. Societally, WPV contributes to mistrust between patients and healthcare workers, economic burden, absenteeism, job burnout and reduced concentration, compromising healthcare delivery and community health [23–29].

To mitigate these adverse consequences, many healthcare institutions worldwide have implemented WPV reporting systems to safeguard their healthcare workers and ensure that assailants are held accountable for their actions. Unfortunately, it was found that only 7.6%–13% of victims formally reporting incidents [30, 31]. Reporting rates remain low across countries, ranged between 11% and 32% [32–34]. Generally, there are three main reasons for underreporting. Firstly, healthcare workers often view violence as part of the job or not serious enough to report, particularly if it involves verbal rather than physical aggression [35–39]. Secondly, lack of clear reporting systems, weak management support, fear of reprisal and perceptions that no action will be taken discourage reporting [36, 40–43]. Thirdly, the time‐consuming and complicated nature of reporting procedures further reduces reporting [36, 44, 45].

Adding to the challenge of underreporting, several problems are known to persist within the existing WPV reporting forms. Firstly, there is no standardised or uniform form that allows consistent data collection and meaningful comparison across different healthcare institutions [46]. Current approaches to measuring WPV prevalence differ considerably, relying on sources such as formal injury records [47], daily ward logs [48] and retrospective reporting [49]. These methods are further complicated by inconsistent definitions of ‘incident’ and ‘injury’, as well as differing reference periods, ranging from ‘past 6 months’ [50] to ‘past 1 year’ [49]. Such variations limit the accuracy of prevalence estimates and make cross‐study comparisons difficult. Secondly, the challenges of WPV reporting were further intensified during the COVID‐19 pandemic, when healthcare workers faced overwhelming workloads, emotional strain and limited organisational support. Those caring for COVID‐19 patients reported significantly higher rates of physical (aOR = 2.18) and verbal (aOR = 2.10) WPV, with 10% indicating that reporting such incidents became even more difficult during this period [51]. Additionally, the structure and content of existing reporting forms vary widely. Some are overly simplistic, containing only three to five items that capture minimal details about the victim or the incident. Conversely, some forms are excessively detailed, requiring completion of hundreds of items, which can lead to respondent fatigue or discourage reporting altogether. Moreover, most existing forms emphasise information about the victim and the incident but fail to capture critical data on the assailant’s characteristics, control measures implemented and postincident prevention and management strategies [52–54]. This lack of comprehensiveness can hinder future data analysis, institutional auditing and evidence‐based policy development.

Given the limitations of existing WPV reporting forms, the present review aimed to identify all available WPV reporting forms used worldwide. Then, it further examined the common domains covered within these forms and to determine the key information that should be included in an effective and comprehensive WPV reporting form.

## 2. Materials and Methodology

### 2.1. Information Sources and Search Strategy

A comprehensive and systematic search strategy was employed to identify literature related to WPV reporting in healthcare settings. The search was designed to be both sensitive and specific to ensure the inclusion of all relevant studies and/or reporting forms. Three electronic databases, namely PubMed, Google Scholar, and Web of Science, were searched for eligible publications.

The search strategy combined both controlled vocabulary and free‐text terms, using the following keywords: violence, harassment, reporting, notification, workplace, hospital, healthcare facility and ward. The specific search strategy for each database is provided in Supporting File 1. To supplement the electronic database search, grey literature was identified through targeted searches of Google Scholar. Additionally, the reference lists of all included studies and relevant reviews were manually screened to capture further publications that might not have been retrieved through the database searches.

### 2.2. Eligibility Criteria

Studies were selected based on predefined inclusion and exclusion criteria. Articles were eligible if they reported on the reporting of WPV in healthcare settings, were published between 1990 and 2025, and were written in English. Only quantitative studies, including cross‐sectional, case–control and randomised controlled trials, were included. Quantitative studies were included because they utilised various questionnaires and survey instruments that contained components comparable to WPV reporting forms. These instruments captured multiple items and domains of WPV incidents, thereby enabling the extraction and identification of relevant reporting items for this review. Grey literature sources relevant to WPV reporting were also considered eligible for inclusion.

Studies were excluded if they focused on WPV involving students or trainees in healthcare settings, violence perpetrated by healthcare workers or violence occurring outside healthcare contexts. In addition, study protocols, conference proceedings and preprints were excluded. Studies using qualitative designs without quantitative data were also excluded from the review.

### 2.3. Selection Process

All retrieved articles were imported into reference management software (Rayyan.AI), and duplicate records were removed prior to screening. Study selection was conducted in two stages. In the first stage, two independent reviewers (Sheng Qian Yew and Mohd Syazmin Zuwairy Mohd Sharial) screened the titles and abstracts of all retrieved records against the eligibility criteria. Any discrepancies between reviewers were resolved through discussion. In cases where consensus could not be reached, a third reviewer (Hanizah Mohd Yusoff) was consulted to make a final decision. In the second stage, the same reviewers independently conducted a full‐text screening of all potentially eligible articles to confirm their inclusion. Disagreements regarding study eligibility were discussed between reviewers, and unresolved disagreements were again referred to the third reviewer for adjudication.

### 2.4. Data Collection Process

A standardised data extraction form was developed to ensure consistency in the collection of relevant study information. For each included study, data were extracted on the following variables: author(s), year of publication, country of study, study design, sample size, study population, name of the WPV reporting tool, number of items in the tool and the reporting approach or system described. All extracted information was recorded systematically using a structured data extraction template to maintain transparency, reproducibility and completeness.

### 2.5. Hierarchical Cluster Analysis

To examine structural similarities among WPV reporting forms, hierarchical cluster analysis was conducted. Each reporting form was converted into a binary matrix in which the presence of each identified item was coded as 1 and absence as 0.

Since the data consisted of binary variables and shared absences were not considered indicative of conceptual similarity, the Jaccard coefficient was used as the measure of proximity. The Jaccard coefficient evaluates similarity based on shared presences while excluding joint absences, making it appropriate for content comparison of structured reporting instruments.

An agglomerative hierarchical clustering procedure was performed using between‐groups average linkage (average linkage between groups) in SPSS. This linkage method was selected to provide balanced clustering and to reduce the chaining effect commonly observed with single linkage methods.

A dendrogram was generated to visualise cluster formation. The number of clusters was determined through visual inspection of the dendrogram, considering substantial increases in rescaled distance coefficients and interpretability of cluster solutions.

Following identification of clusters, comparative analyses were performed to characterise cluster profiles. For each cluster, the following characteristics were examined for the number of items and key domains represented.

## 3. Results

**FIGURE 1:**
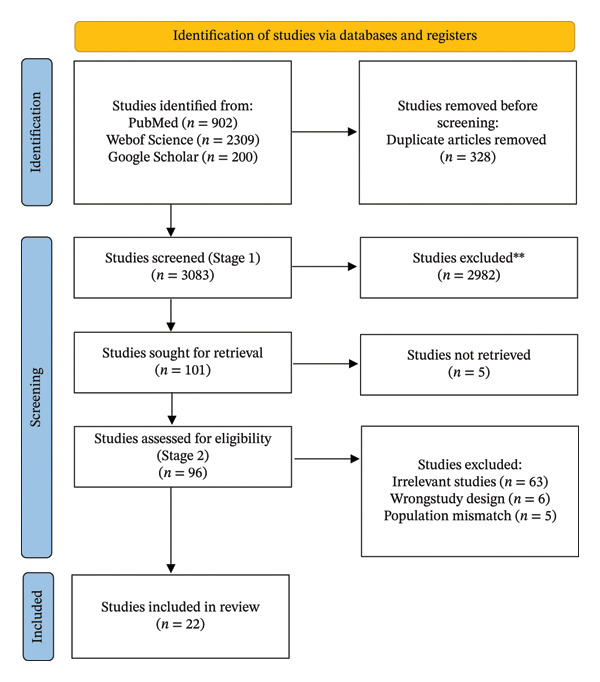
PRISMA flowchart of this review.

A total of 3411 studies were identified through the database search, including PubMed (*n* = 902), Google Scholar (*n* = 200), and Web of Science (*n* = 2309). After removing 328 duplicate studies, 3083 studies remained for title and abstract screening. Following this initial screening (Stage 1), 2982 studies were excluded due to irrelevance to the research topic, leaving 101 studies for full‐text retrieval. Full texts could not be obtained for five studies, resulting in 96 studies assessed for eligibility. During the eligibility assessment (Stage 2), 74 studies were excluded for reasons such as irrelevant studies (*n* = 63), wrong study design (*n* = 6) and population mismatch (*n* = 5). Ultimately, 22 studies met the inclusion criteria and were included in the systematic review. This is illustrated in Figure 1.

A total of 22 studies (or WPV reporting forms) were included in this review (Table 1). Of these, 11 originated from North America, five from Australasia, four from Europe, one from South Africa and one involved multiple countries. In terms of study characteristics, the majority of studies adopted a cross‐sectional design (*n* = 11), followed by mixed‐methods studies (*n* = 3), secondary analyses (*n* = 2) and randomised controlled trials (*n* = 1). In five studies, the design was not applicable as they are solely WPV forms rather than empirical studies. The number of items across the reporting forms ranged widely from 3 to 114. Most of the reporting forms were available in physical format (*n* = 12), while six were online‐only, two used mixed formats, one was a mobile application and one did not specify the format used.

**TABLE 1:** Characteristics of the included studies (or reporting form).

Authors and years	Country	Study designs	Sample size	Study populations	Name of reporting tools	No. of items	Approach of reporting
Arnetz 1998 [55]	• Sweden	• Cross‐sectional study	• 47 centres (with 684 incidents)	• All healthcare workers in hospital	• Violence incidence form checklist	• 23	• Physical form
Arnetz 2011 [56]	• United States	• Cross‐sectional study	• 1126 incidents (over 6 years in 6 hospitals)	• All healthcare workers in hospital	• Workplace violence incident reports	• 23	• Physical and online form
Bowers et al. 2005 [48]	• United Kingdom	• Cross‐sectional study	• 13 psychiatric centres (with 15,006 incidents across 2 years)	• Nurses in psychiatry wards	• Patient–staff conflict checklist shift report (PCC‐SR)	• 3	• Physical form
Byon et al. 2022 [51]	• United States	• Cross‐sectional study	• 373 participants (with 742 incidents)	• Nurses	• Type II workplace violence reporting form	• 10	• Online form
Calik et al. 2021 [57]	• Türkiye	• Secondary data analysis	• 315 participants (with 316 incidents across 4 years)	• All healthcare workers in hospital	• White code incident form	• 12	• Physical form
California Hospital Association 2019 [58]	• United States	• NA	• NA	• All healthcare workers in hospital	• Workplace violence incident form	• 21	• Physical form
Cikriklar et al. 2016 [59]	• Türkiye	• Cross‐sectional study	• 239 incidents	• All healthcare workers in emergency department	• Emergency department violence questionnaire	• 13	• Physical form
Colorado Hospital Association 2022 [60]	• United States	• NA	• NA	• All healthcare workers in hospital	• Incident review form	• 14	• Physical form
Hamblin et al. 2017 [61]	• United States	• Randomised controlled trial	• 32 participants	• All healthcare workers in hospital	• Workplace violence form	• 6	• Online form
Health Professionals and Allied Employees 2016 [62]	• United States	• NA	• NA	• All healthcare workers in hospital	• HPAE workplace violence reporting form	• 18	• Physical form
Khedr et al. 2024 [63]	• Egypt	• Cross‐sectional study	• 250 participants	• Physicians only	• NA	• 25	• Online form
Kim and Kim 2023 [64]	• United States	• Cross‐sectional study	• 96 participants	• All healthcare workers in hospital	• Violent event severity tool (VEST)	• 14	• NA
Massachusetts Nurses Association 2022 [65]	• United States	• NA	• NA	• All healthcare workers in hospital	• MNA workplace violence reporting form	• 19	• Physical form
McGuire et al. 2021 [50]	• United States	• Cross‐sectional study	• 242 participants	• All healthcare workers in emergency department	• NA	• 10	• Online form
Ministry of Health Malaysia 2022 [66]	• Malaysia	• NA	• NA	• All healthcare workers in hospital	• Violence reporting Forms 1 and 2	• 37	• Physical form
Odes et al. 2022 [67]	• United States	• Cross‐sectional study	• 413 facilities	• All healthcare workers in hospital	• Workplace violent incident reporting system (WVIRS)	• 20	• Online form
Olabisi et al. 2025 [68]	• Nigeria	• Mixed‐methods study	• 401 participants	• All healthcare workers in hospital and clinics	• Workplace violence questionnaire (WVQ)	• 36	• Physical form
Pompeii et al. 2016 [69]	• United States	• Mixed‐methods study	• 6 hospitals, 11,000 participants	• All healthcare workers in hospital	• NA	• 16	• Physical and online form
Ramacciati et al. 2021 [70]	• Italy	• Cross‐sectional study	• 184 participants	• Nurses	• PSaggress app	• 5	• Mobile application
Renwick et al. 2016 [47]	• United Kingdom	• Secondary data analysis	• 552 incidents	• All healthcare workers in mental health centres	• Reporting of injuries, diseases and dangerous occurrences regulations form	• 28	• Online form
Richardson et al. 2018 [71]	• New Zealand	• Cross‐sectional study	• 107 participants	• All healthcare workers in emergency department	• NA	• 7	• Physical form
WHO 2003 [49]	• Multiple	• Mixed‐methods study	• 6099 participants	• All healthcare workers in hospital	• Workplace violence in the health sector country case studies research questionnaire	• 114	• Physical form

Most studies presented their sample size based on the number of participants, which ranged from 32 to 6099 individuals. A few studies, however, reported their sample size in terms of healthcare facilities, ranging from 13 to 413 facilities. Some others described the sample size by the number of reported WPV incidents, which varied between 239 and 552 cases. The majority of studies were conducted among hospital healthcare workers (*n* = 18), while a smaller number specifically focused on nurses (*n* = 3) or physicians (*n* = 1) (Table 1).

Analysis of the 22 studies (or WPV reporting forms) identified a total of 13 domains encompassing 148 distinct items related to WPV reporting (Table 2). The first domain, sociodemographic data of the victim (11 items), typically included variables such as age, gender and ethnicity. The job characteristics of the victim domain (29 items) was among the most comprehensive, covering aspects such as job title, department, employment status, shift schedule and duration of working experience. The characteristics of the notifier domain (4 items) focused on identifying who reported the incident, their job title and their contact information.

**TABLE 2:** Domains and items of the included studies (or reporting forms) and their respective frequencies.

Domains	Items	Frequencies (%)
Sociodemographic data of the victim	Name of the victim	5 (22.7)
	Identification card number of the victim	1 (4.5)
	Contact number of the victim	2 (9.1)
	Gender of the victim	14 (63.6)
	Age of the victim	11 (50.0)
	Nationality of the victim	2 (9.1)
	Date of arrival in the country of the victim (for foreigner)	1 (4.5)
	Ethnicity of the victim	5 (22.7)
	Marital status of the victim	2 (9.1)
	Working address of the victim	1 (4.5)
	Religion of the victim	1 (4.5)

Job characteristics of the victim	Job position of the victim	15 (68.2)
	Job title of the victim	2 (9.1)
	Department (or speciality) of the victim	5 (22.7)
	Victim’s mode of working (shift)	1 (4.5)
	Primary working shift of the victim	1 (4.5)
	Work in shift	2 (9.1)
	Number of workers on duty in every shift	2 (9.1)
	Number of incidents in every shift	1 (4.5)
	Number of containments in every shift	1 (4.5)
	Qualification of the victim	3 (13.6)
	Speciality of the victim	1 (4.5)
	Place of employment of the victim	2 (9.1)
	Employment status of the victim	2 (9.1)
	Name of the hospital	1 (4.5)
	Regions of hospital	1 (4.5)
	Level of healthcare (primary, secondary, tertiary)	2 (9.1)
	Supervisor status	1 (4.5)
	Name of the supervisor	1 (4.5)
	Duration of working experience in healthcare of the victim	8 (36.4)
	Duration of working experience in the hospital of the victim	2 (9.1)
	Duration working in emergency department of the victim	1 (4.5)
	Previous experience (frequency) of violence	9 (40.9)
	Paid productive hours (PPH) of the victim	1 (4.5)
	Victim’s routine direct physical contact with patients	1 (4.5)
	Types of patients most frequently work with	1 (4.5)
	No. of staff present in the same work setting with victim	1 (4.5)
	Location where victim spend most of the time	1 (4.5)
	Experience more violence than before COVID‐19	1 (4.5)
	Gender of patients whom victim most frequently works with	2 (9.1)

Characteristics of the notifier	Name of the notifier	2 (9.1)
	Job position of the notifier	1 (4.5)
	Work address of the notifier	1 (4.5)
	Contact number of the notifier	1 (4.5)

Characteristics of the incident	Date of the incident	9 (40.9)
	Year of the incident	3 (13.6)
	Season of the incident	1 (4.5)
	Month of the incident	1 (4.5)
	Day (of the week) when the incident occurs	3 (13.6)
	Time of the incident	15 (68.2)
	Work shift in which the incident occurs	1 (4.5)
	Local authority under which the incident occurs	1 (4.5)
	Types of industry which the incident occurs	1 (4.5)
	Hospital where the incident occurs	3 (13.6)
	Address of the hospital	2 (9.1)
	Location (department) of the incident	13 (59.1)
	Instrument (weapon) used in the violence	7 (31.8)
	Main activity at the workplace	1 (4.5)
	Types of violence	14 (63.6)
	Description of the incident	8 (36.4)
	Reason(s) of violence	6 (27.3)
	Severity of the violence	2 (9.1)
	Manner(s) of attack	1 (4.5)
	Activities preceding or leading to the incident	3 (13.6)
	Activities during the incident	4 (18.2)
	Environmental conditions at the time of the incident	1 (4.5)
	Feeling of the victim in advance	1 (4.5)
	Victim was alone or in an isolated area	3 (13.6)
	Victim work in a location that was unfamiliar or new	1 (4.5)
	Victim is performing an unfamiliar or new task	1 (4.5)
	Duration of incident from the commencement of shift	2 (9.1)
	Other nonhospital staff affected	1 (4.5)
	Continuing threats to the victim	1 (4.5)

Characteristics of the injury	Part(s) of the body attacked or injured	2 (9.1)
	Injury status of the victim	9 (40.9)
	Types of injury sustained by the victim	2 (9.1)
	Severity of injury of the victim	2 (9.1)
	Victim deceased as a result of the injury	1 (4.5)
	Injury status of the assailant	1 (4.5)
	Severity of injury of the assailant	1 (4.5)
	Number of staffs injured	1 (4.5)

Measures taken during the violence	Action (or reaction) taken	5 (22.7)
	Personnel who took the action or provide assistance	3 (13.6)
	Risk reduction measures toward the assailant	1 (4.5)
	Status of implementation of appropriate measures	3 (13.6)
	Status of availability of alarm (or other assistance) during the incident	1 (4.5)
	Use alarm (or other assistance) during the incident	2 (9.1)
	Response of staff or law enforcer	1 (4.5)
	Enforcement of law or legal outcome	3 (13.6)
	Status of police report	2 (9.1)
	Termination of the incident	1 (4.5)
	Use of restrain	1 (4.5)
	Assistance or support provided by employer or supervisor	3 (13.6)
	Organisation has a violence prevention program	3 (13.6)
	Presence of violence policies at workplace	1 (4.5)

Consequences of the violence	Immediate health effects (consequence) of the violence to the victim	2 (9.1)
	Long‐term consequences of the violence to the victim	1 (4.5)
	Time lost due to the violence	1 (4.5)
	Take time off from work after the incident	5 (22.7)
	Number of days victim take time from work	2 (9.1)
	Consequences to the assailant	3 (13.6)

Postviolence treatment	Treatment given to the victim	4 (18.2)
	Debriefing given to the victim	1 (4.5)
	Mental health assessment to the victim after the incident	1 (4.5)
	Referral of the victim to counsellor	1 (4.5)
	Referral of the victim to psychiatry	1 (4.5)

Aftermath of the violence	Measures taken to prevent future violence	3 (13.6)
	Action taken to investigate the causes of the violence	2 (9.1)
	Suggestion of measures to prevent future violence	9 (40.9)
	Implementation of changes in the workplace	1 (4.5)
	Impact of the changes on daily work	1 (4.5)

Reporting of the violence	Status of reporting the incident	5 (22.7)
	Personnel to whom the violence was reported	1 (4.5)
	Reporting date	3 (13.6)
	Reporting time	2 (9.1)
	Presence of reporting procedure of violence in the workplace	2 (9.1)
	Familiarity of reporting	2 (9.1)
	Previous reporting of violence	2 (9.1)
	Frequency of reporting violence	1 (4.5)
	Reporting of violence using another approach	2 (9.1)
	Reason for not reporting violence	4 (18.2)
	Presence of encouragement to report violence in the workplace	1 (4.5)
	Personnel who encourage the reporting of violence	1 (4.5)
	Disciplinary action taken for reporting workplace violence	1 (4.5)
	Difficulty of reporting violence than before COVID‐19	1 (4.5)

Perception of victim postviolence	Perceived cause of the increasing violence	2 (9.1)
	Perception that the violence could have been prevented	3 (13.6)
	Perception that violence is typical in the workplace	1 (4.5)
	Perception that preventive measures would be helpful	2 (9.1)
	Attitude of victim following the incident	2 (9.1)
	Perceptions of safety at the workplace	3 (13.6)
	Preference to be contacted for further assistance	1 (4.5)
	Level of worry about violence	2 (9.1)
	Level of satisfaction of the victim with the manner in which the violence was handled	2 (9.1)
	Perceived intent to harm	1 (4.5)

Characteristics of the assailant	Age of the assailant	2 (9.1)
	Gender of the assailant	2 (9.1)
	Assailant’s patient registration number	1 (4.5)
	Typology of the assailant	14 (63.6)
	Conditions or characters of the assailant	2 (9.1)
	Assailant’s risk factors	4 (18.2)
	Assailant’s history of violence	1 (4.5)
	Disposition of the assailant	2 (9.1)
	Relationship of the assailant to the victim	1 (4.5)

Characteristics of the witness	Availability of witness	3 (13.6)
	Name of the witness	3 (13.6)
	Job title of the witness	1 (4.5)
	Contact of witness	1 (4.5)
	Frequency of witnessing physical violence	1 (4.5)

Another key domain was the characteristics of the incident (29 items), which captured essential details such as the date, time and location of incident, the type of violence experienced and the activities leading to the event. The characteristics of the injury domain (8 items) included the nature, severity and site of injury, as well as the number of staffs injured. For the measures taken during the violence domain (14 items), it focused on the action or response taken, who took these actions and the availability of violence prevention program in the organisation.

Postincident responses were represented through several domains. The consequences of the violence (6 items) and postviolence treatment (5 items) domains described the effects of the violence on the victim and the treatment received by the victim, respectively. The aftermath of the violence domain (5 items) covered measures taken to prevent future violence and the investigation of the causes of the violence. The reporting of the violence domain (14 items) dealt with procedural aspects, including whether the incident was officially reported, to whom it was reported and the date and time of reporting. The perception of the victim postviolence domain (10 items) explored the victim’s emotional response, perceived safety and satisfaction with the handling of the incident. Information on the perpetrator was recorded in the characteristics of the assailant domain (9 items), covering demographic background, typology and the existing risk factors on them. Finally, the characteristics of the witness domain (5 items) described the presence and involvement of witnesses and their names and contact information. The full item lists of each WPV reporting tool are available in Supporting File 2.

Although no standard currently defines which items are essential in reporting forms, several items were commonly reported across the reviewed forms. The most commonly included items across the reviewed forms were job position of the victim (68.2%), time of the incident (68.2%), gender of the victim (63.6%), types of violence (63.6%), typology of the assailant (63.6%), location or department of the incident (59.1%), age of the victim (50.0%), injury status of the victim (40.9%) and suggested measures to prevent future violence (40.9%). Overall, the reviewed reporting forms demonstrated substantial variability in structure, content and comprehensiveness, reflecting the absence of a standardised framework for WPV reporting in healthcare settings. The detailed domains and items of each WPV reporting form are available in Supporting File 3.

Pairwise structural similarity among the 22 studies (or WPV reporting forms) was assessed using Jaccard coefficients based on 148 dichotomously coded items. The Jaccard similarity coefficients ranged from 0.00 to 0.53, with a mean similarity of 0.14 (SD = 0.09), indicating substantial heterogeneity in item coverage across the WPV reporting forms.

Agglomerative hierarchical clustering using average linkage (between‐groups linkage) was performed. Inspection of the agglomeration schedule revealed a marked increase in the coefficient between Stages 20 and 21, suggesting that substantially dissimilar clusters were merged at this stage (Supporting File 4).

**FIGURE 2:**
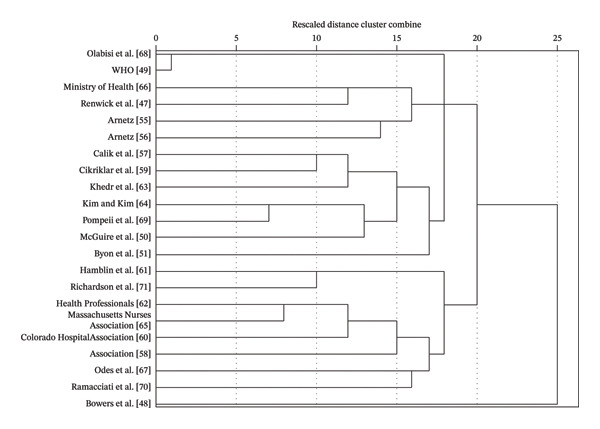
Dendogram using average linkage (between groups).

Based on this discontinuity and visual inspection of the dendrogram (Figure 2), a three‐cluster solution was considered optimal. Given that there was no existing literature that proposed a classification of the WPV reporting form, for the purpose of this review, these three clusters were named as rapid, brief and detailed WPV reporting forms.

WPV reporting forms in Cluster 1 are known as rapid WPV reporting forms (*n* = 1). They contained the lowest number of items (3 items) and were largely restricted to basic incident reporting with job characteristics of the victim domain. WPV reporting forms in Cluster 2 are known as brief WPV reporting forms (*n* = 8). They have moderate coverage (mean = 13.75 items) that emphasised on seven domains such as job characteristics of the victim, characteristics of the incident, characteristics of the injury, measures taken during the violence, characteristics of assailant, characteristics of the witness and reporting of the violence. For Cluster 3 (known as detailed WPV reporting form) (*n* = 13), it has high coverage (mean = 27.77 items) and exhibited broad domain coverage, with the inclusion of all 13 domains in most cases. These forms consistently captured domains such as sociodemographic data of the victim, job characteristics of the victim, characteristics of the notifier, characteristics of the incident, characteristics of the injury, measures taken during the violence, consequences of the violence, postviolence treatment, aftermath of the violence, reporting of the violence, perception of the victim postviolence, characteristics of the assailant, and characteristics of the witness. These are summarised in Table 3 below.

**TABLE 3:** Summary of domains in each cluster of WPV reporting form.

Cluster 1 (rapid WPV reporting form)	Cluster 2 (brief WPV reporting form)	Cluster 3 (detailed WPV reporting form)
Job characteristics of the victim		
	Characteristics of the incident	
	Characteristics of the injury	
	Measures taken during the violence	
	Characteristics of assailant	
	Characteristics of the witness	
	Reporting of the violence	
		Sociodemographic data of the victim
		Characteristics of the notifier
		Consequences of the violence
		Postviolence treatment
		Aftermath of the violence
		Perception of the victim postviolence

The labels ‘rapid’, ‘brief’, and ‘detailed’ were assigned post hoc based on the observed range of item counts within each cluster to aid interpretability. However, to avoid overinterpretation, we have revised the manuscript to clarify that these are descriptive labels rather than analytically derived categories. Based on the current literature, there is no established cutoff point for the number of items used to classify WPV reporting forms into distinct categories.

## 4. Discussion

The job characteristics of the victim domain was given much attention across all clusters of WPV reporting forms in healthcare settings because this domain is strongly associated with the risk and exposure of WPV. Of note, information such as job title was the most frequently asked items under this domain (Table 2). This is because healthcare professionals of different job roles face varying levels of exposure to patients, relatives or the public. For instance, nurses, emergency staff and psychiatric unit workers often encounter higher risks of WPV due to their frequent and direct contact with patients in stressful or unpredictable situations [72, 73]. The secondly asked item under this domain was the previous experience of violence. This item is linked to both coping and reporting behaviours, whereby workers who have not experienced WPV may have lower confidence or skills in de‐escalating aggressive situations and might also be less likely to report incidents [74].

Characteristics of the incident was another domain that was given much attention in WPV reporting forms because it provides critical contextual information that helps researchers and institutions understand how, when and why violent incidents occur. Firstly, documenting the date and time of incidents helps to identify temporal patterns of violence. For example, certain times of the day, such as night shifts or weekends, may be associated with higher risks due to reduced staffing, increased patient waiting times or higher stress levels [75]. Secondly, recording the location of the incident provides insights into where WPV most frequently occurs within healthcare facilities. High‐risk areas such as emergency departments, psychiatric wards and outpatient clinics often experience more incidents due to high patient volumes, emotional distress or unpredictable behaviours [76]. Thirdly, the type of violence experienced also plays a vital role in understanding the severity and nature of WPV. Different types of violence may require different preventive measures and training approaches [49].

On top of these, the characteristics of the injury domain cannot be omitted because it provides information on the nature, severity and site of injuries, as well as the number of staffs affected. Specifically, the item of injury status of the victim is particularly helpful to quantify the physical impact of WPV and guide the allocation of medical resources, compensation claims and occupational safety measures [77]. Likewise, the measures taken during the violence domain is equally important, as it highlights the immediate actions or responses taken during the incident, such as calling for security, de‐escalation attempts or evacuation [78]. The consequences of the violence and postviolence treatment domains focus on the short‐ and long‐term effects of WPV on victims, including physical, psychological and occupational outcomes, as well as the medical or psychological treatment provided. These domains are to ensure that affected employees receive appropriate care and rehabilitation [79].

The characteristics of the assailant domain allows the understanding of who and under what conditions the assailant commits violence. Specifically, the item of typology of assailant enables institutions to identify high‐risk individuals so as to develop targeted prevention strategies, such as flagging the patients who could potentially cause WPV [80].

The characteristics of the witness domain is also vital, as it captures information about who observed the incident and their level of involvement. Witnesses can provide independent, objective accounts that enhance the accuracy and credibility of WPV reports, especially when victims are too distressed or reluctant to report details themselves [81].

The reporting of the violence domain deals with the procedural and administrative aspects of how WPV incidents are communicated within the organisation. Particularly, capturing item about status of reporting the incident provides insights into reporting culture, timeliness and compliance with institutional policies [82]. Low reporting rates or delays may indicate barriers such as fear, lack of awareness or inefficiencies in the reporting process that require organisational attention.

Having said the above, although other domains were given less attention, these domains were not ‘unimportant’ in WPV reporting because they do help to capture the full spectrum of the incident. For instance, the sociodemographic data of the victim domain cannot be missed because individual factors such as age, gender and ethnicity can influence both the risk of experiencing WPV and the way victims respond to it. For example, younger or less experienced healthcare workers may be more susceptible to verbal or physical aggression due to lower authority or confidence in handling difficult patients [83]. Gender differences may also shape the type of violence encountered. Female staff, for instance, may face higher risks of sexual harassment and verbal violence [84], while male staff may experience more physical aggression [85]. Ethnicity or cultural background can also hinder communication and perceived respect and eventually reduce the likelihood of reporting incidents [86]. In a recent study, healthcare workers belonging to ethnic minority were more likely to experience WPV [87]. Collecting sociodemographic data enables organisations to identify at‐risk populations and design targeted prevention programs that are equitable and culturally sensitive.

Of note, the characteristics of the notifier domain received the least attention in WPV reporting forms. This is because it is often viewed as administratively secondary compared to information about the incident itself or the victim. In many cases, the primary objective of WPV reporting is to document what happened, who was affected and under what circumstances, rather than focussing on the individual who submitted the report. Additionally, the notifier is often the same person as the victim, especially in self‐reported cases. In such situations, the notifier’s information would be redundant, as their job title and position are already captured under the job characteristics of the victim domain. Therefore, many forms, especially from Cluster 1, omit or simplify this domain to avoid duplication [47, 69, 71].

The aftermath of the violence domain plays a preventive role by documenting organisational follow‐up actions, such as investigations, root cause analyses and preventive measures implemented after the event [88].

The perception of the victim postviolence domain is particularly important in understanding the psychological aftermath and satisfaction with the institutional response. These insights are essential for evaluating whether postincident management is victim‐centred and whether additional support mechanisms are sufficient to promote recovery and workplace trust [89].

### 4.1. Strengths and Limitations of the Review

This systematic review possesses several notable strengths. To the best of current knowledge, it is the first review to comprehensively identify and analyse all available WPV reporting forms used in healthcare settings. Secondly, the review employed a broad and inclusive search strategy across multiple databases including grey literature to ensure that relevant forms and studies were captured, regardless of publication type or region. Thirdly, the review systematically categorised forms according to key domains such as victim characteristics, incident details and postincident responses. This provides valuable insights into the completeness and variability of WPV documentation. Additionally, by incorporating studies from diverse healthcare systems worldwide, the review offers a global perspective on reporting practices and highlights both common features and gaps across different contexts.

However, several limitations should also be acknowledged. The review may be subject to publication bias, as some relevant WPV reporting forms, such as the Notification of Workplace Violence Form and California Workplace Violence Incident Form have not undergone empirical studies and have not been published. Not to mention, the content of these reporting forms was not assessed of their psychometric properties (e.g., validity and reliability) and overall quality. Moreover, traditional quality assessment tools could not be fully applied since many of the included materials were reporting forms or descriptive documents rather than empirical research studies. Besides this, considerable heterogeneity was also observed among the included forms and studies in terms of structure, purpose and context, which limits the ability to compare findings directly across different settings.

Another limitation is that the review focused primarily on the content of the forms rather than their effectiveness, utilisation or impact on reporting behaviour and workplace safety outcomes. Despite these limitations, this systematic review provides a strong foundation for improving WPV reporting systems and supports the development of standardised, comprehensive and user‐friendly reporting practices in healthcare. Additionally, this review was limited to WPV reporting forms used among employed healthcare workers in formal healthcare workplace settings and did not include instruments designed for healthcare students in academic or clinical training environments. This exclusion was due to differences in governance, structure and reporting pathways between occupational reporting systems and student‐based reporting mechanisms. Finally, some internally used WPV reporting forms may not be publicly accessible (i.e., indexed in electronic databases). Even if they were reported, only English‐language publications were included in this review.

### 4.2. Implications of the Current Review

This systematic review has several important implications for occupational health practice, policy and research in addressing WPV in healthcare settings. The review highlights significant variability in the content, structure and domains of existing WPV reporting forms. This underscores the urgent need to develop standardised, evidence‐based reporting tools that capture essential information across all key domains in clinical practice. Standardisation would enhance the comparability of data across institutions and countries, enabling more accurate benchmarking and global surveillance of WPV in healthcare. Although this review identified a total of 148 items, it is not necessary for all of them to be incorporated into a WPV reporting form. It is recommended that, at a minimum, the top seven most frequently reported items (as outlined in Table 3) be included in any WPV reporting tool to ensure essential information is captured. This is to prevent respondent fatigue while administering the form.

In terms of occupational health policy, health authorities and hospital administrators can use the findings to review and update institutional WPV policies, ensuring that reporting procedures are clear, accessible and aligned with international best practices. The inclusion of domains related to prevention programs, incident management and aftermath measures can also guide policy reforms and resource allocation to strengthen safety infrastructure.

In terms of occupational health research, future studies should assess how well these WPV tools capture real‐world incidents, influence reporting behaviour and contribute to reducing WPV prevalence. Cross‐cultural validation of standardised forms would further ensure their applicability in diverse healthcare contexts.

## 5. Conclusion

This review identified 22 WPV reporting forms comprising 148 items across 13 domains. The forms could be broadly categorised into three groups: rapid, brief and detailed reporting tools. Despite this variability, seven commonly reported domains were consistently identified across the included forms, including job characteristics of the victim, characteristics of the incident, characteristics of the injury, measures taken during the violence, characteristics of the assailant, characteristics of the witness and reporting of the violence. These findings provide a descriptive overview of existing WPV reporting structures and may serve as a reference for future development of reporting tools. However, the study does not assess the effectiveness or validity of these domains, and their inclusion in future forms should be considered in relation to context, feasibility and implementation needs.

### 5.1. Implication for Nursing Management

In terms of nursing practice, the review highlights significant variability in the content, structure and domains of existing WPV reporting forms used in healthcare institutions. This underscores the urgent need to develop standardised, evidence‐based reporting tools that capture essential information across all key domains relevant to nursing practice. Standardisation would enhance the comparability of data across institutions and countries, enabling more accurate benchmarking and global surveillance of WPV in healthcare settings where nurses represent the largest proportion of frontline staff. Although this review identified a total of 148 items, it is not necessary for all of them to be incorporated into a WPV reporting form. It is recommended that, at a minimum, the top seven most frequently reported items (as outlined in Table 3) be included in any WPV reporting tool to ensure that essential information is captured while preventing respondent fatigue among nurses completing the form.

In terms of nursing policy, nursing leaders, hospital administrators and health authorities can use these findings to review and strengthen institutional WPV policies. Clear, accessible and standardised reporting procedures can encourage nurses to report incidents more consistently and accurately. In addition, incorporating domains related to prevention programs, incident management and postincident support can guide policy development, staff training initiatives and resource allocation to enhance safety within nursing practice environments.

In terms of nursing research, future studies should examine how effectively these WPV reporting tools capture real‐world incidents involving nurses, influence reporting behaviour among nursing staff and contribute to reducing the prevalence of WPV in healthcare settings. Furthermore, cross‐cultural validation of standardised WPV reporting forms is needed to ensure their applicability across different healthcare systems and nursing contexts worldwide.

## Author Contributions

Sheng Qian Yew and Hanizah Mohd Yusoff conceptualised and designed the study. Sheng Qian Yew and Mohd Syazmin Zuwairy Mohd Sharial performed data collection and data analysis. Sheng Qian Yew drafted the manuscript, and all authors critically revised the manuscript for intellectual content.

## Funding

This research received funding from the Faculty of Medicine, National University of Malaysia (FF‐2025‐265) by Sheng Qian Yew.

## Disclosure

All authors have read and approved the final version of the manuscript and agree to be accountable for all aspects of the work.

## Ethics Statement

Ethical approval was not required for this review.

## Conflicts of Interest

The authors declare no conflicts of interest.

## Supporting Information

Additional supporting information can be found online in the Supporting Information section.

## Supporting information


**Supporting Information 1** Supporting File 1: Search strategy for each database.

**Supporting Information 2** Supporting File 2: Full item lists of each WPV reporting tool.

**Supporting Information 3** Supporting File 3: Detailed domains and items of each WPV reporting form.

**Supporting Information 4** Supporting File 4: Agglomeration schedule.

## Data Availability

Data will be available upon reasonable request.
