# Supplementary material for: How to Adequately Report Workplace Violence in Healthcare Setting: A Systematic Review With Hierarchical Cluster Analysis of Workplace Violence Reporting Forms
Source: J Nurs Manag. 2026 Jun 28;2026:4803748. doi: 10.1155/jonm/4803748 (PMC13310381; doi:10.1155/jonm/4803748)
Supplement: Supplementary file 1 — Supporting Information 1 Supporting File 1: Search strategy for each database. [file JONM-2026-4803748-s002.docx]

Scopus:

(Violen* OR harass*) AND (Report* OR Notif*) AND (Hospital OR healthcare facility OR ward)

Web of science:

TS=(Violen* OR harass*) AND TS=(Report* OR Notif*) AND TS=(Hospital OR “healthcare facility” OR ward)

Google Scholar (include only the first 20 pages, which consists of 200 articles):

(violence OR harassment) AND (reporting OR notification) AND (hospital OR “healthcare facility” OR ward)
