# Supplementary material for: How to Adequately Report Workplace Violence in Healthcare Setting: A Systematic Review With Hierarchical Cluster Analysis of Workplace Violence Reporting Forms
Source: J Nurs Manag. 2026 Jun 28;2026:4803748. doi: 10.1155/jonm/4803748 (PMC13310381; doi:10.1155/jonm/4803748)
Supplement: Supplementary file 2 — Supporting Information 2 Supporting File 2: Full item lists of each WPV reporting tool. [file JONM-2026-4803748-s003.docx]

| **Authors, Years, and Countries** | **Study Designs and Sample Size** | **Name of Reporting Tools, No. of Items, and Approach of Reporting** | **Items and Response Options** |
| --- | --- | --- | --- |
| Arnetz, 1998  Country: Sweden | - Study design: Cross-sectional study - Sample size: 47 centers (with 684 incidents) | - Name of reporting tool: Violence Incidence Form Checklist - No. of items: 23 - Approach of reporting: Physical form | - Name of the victim – Open-ended - Gender of the victim – Multiple choice - Age of the victim – Multiple choice - Job position of the victim – Multiple choice and open-ended - Place of employment of the victim – Open-ended - Date of the incident – Open-ended - Time of the incident – Multiple choice - Location of the incident – Multiple choice and open-ended - Activities preceding the incident – Multiple choice and open-ended - Activities during the incident – Multiple choice and open-ended - Feeling of victim in advance – Multiple choice - Status of working alone – Multiple choice - Duration of incident from the commencement of shift – Multiple choice - Types of violence – Multiple choice and open-ended - Typology of the assailant – Multiple choice and open-ended - Assailant’s patient registration number – Open-ended - Conditions of assailant – Multiple choice - Gender of assailant – Multiple choice - Age of assailant – Multiple choice - Action taken – Multiple choice - Consequences to the victim – Multiple choice and open-ended - Status of police report – Multiple choice - Reporting date – Open-ended |
| Arnetz, 2011  Country: United States | - Study design: Cross-sectional study - Sample size: 1126 incidents (over 6 years in 6 hospitals) | - Name of reporting tool: Workplace violence incident reports - No. of items: 23 - Approach of reporting: Physical and online form | - Gender of the victim – Binary - Age of the victim – Open-ended - Place of employment of victim – Multiple choice - Job position of the victim – Multiple choice - Supervisor status – Binary - Duration of working experience in healthcare of the victim – Open-ended - Duration of working experience in the hospital of the victim – Open-ended - Previous experience of violence in the past 1 year – Multiple choice - Employment status of the victim – Multiple choice - Paid productive hours (PPH) of the victim – Unknown - Types of violence – Multiple choice - Activity preceding the incident – Open-ended - Injury status of the victim – Multiple choice - Time lost due to the violence – Binary - Familiarity of reporting – Binary - Previous reporting of violence – Multiple choice and open-ended - Reporting of violence using another approach – Multiple choice and open-ended - Instrument (weapon) used in the violence – Open-ended - Names of the witness – Open-ended - Date of the incident – Open-ended - Time of the incident – Open-ended - Work shift in which the incident occurs – Unknown - Location of the incident – Multiple choice |
| Bowers et al, 2005  Country: United Kingdom | - Study design: Cross-sectional study - Sample size: 13 psychiatric centers (with 15,006 incidents across 2 years) | - Name of reporting tool: Patient–staff Conflict Checklist Shift Report (PCC-SR) - No. of items: 3 - Approach of reporting: Physical form | - Number of workers on duty in every nurse shift – Open-ended - Number of incidents in every nurse shift – Open-ended - Number of containments in every nurse shift – Open-ended |
| Byon et al, 2022  Country: United States | - Study design: Cross-sectional study - Sample size: 373 participants (with 742 incidents) | - Name of reporting tool: Type II Workplace Violence Reporting Form - No. of items: 10 - Approach of reporting: Online form | - Age of the victim – Open-ended - Gender of the victim – Binary - Ethnicity of the victim – Multiple choice - Duration of working experience of the victim – Open-ended - Department of the victim – Multiple choice - Regions of hospital – Multiple choice - Frequency of physically violence since COVID-19 pandemic – Multiple choice - Frequency of verbal abuse since COVID-19 pandemic – Multiple choice - Experience more violence than before COVID-19 – Multiple choice - Difficulty of reporting violence than before COVID-19 – Multiple choice |
| Calik et al, 2021  Country: Türkiye | - Study design: Secondary data analysis - Sample size: 315 participants (with 316 incidents across 4 years) | - Name of reporting tool: White Code Incident Form - No. of items: 12 - Approach of reporting: Physical form | - Age of the victim – Open-ended - Gender of the victim – Binary - Job position of the victim – Multiple choice - Area of the incident – Multiple choice - Department of the incident – Multiple choice - Types of violence – Multiple choice - Time of the incident – Open-ended - Month of the incident – Open-ended - Season of the incident – Open-ended - Year of the incident – Open-ended - Reasons of violence – Multiple choice - Typology of assailant – Multiple choice |
| California Hospital Association, 2019  Country: United States | - Study design: NA - Sample size: NA | - Name of reporting tool: Workplace Violence Incident Form - No. of items: 21 - Approach of reporting: Physical form | - Name of the victim – Open-ended - Job position of the victim – Open-ended - Contact number of the victim – Open-ended - Date of the incident – Open-ended - Time of the incident – Open-ended - Location of the incident – Open-ended - Description of the incident – Open-ended - Reporting date – Open-ended - Reporting time – Open-ended - Assailant’s risk factors – Open-ended - Assailant’s history of violence – Open-ended - Risk reduction measures towards the assailant – Open-ended (2 items) - Status of implementation of appropriate measures – Binary and open-ended - Status of availability of alarm (or other assistance) during the incident – Open-ended - Use alarm (or other assistance) during the incident – Open-ended - Response of staff or law enforcer – Open-ended - Victim’s perceived cause of violence – Open-ended - Victim’s suggestion of preventive measure – Open-ended - Witness’ perceived cause of violence – Open-ended - Witness’ suggestion of preventive measure – Open-ended |
| Cikriklar et al, 2016  Country: Türkiye | - Study design: Cross-sectional study - Sample size: 239 incidents | - Name of reporting tool: Emergency Department Violence Questionnaire - No. of items: 13 - Approach of reporting: Physical form | - Gender of the victim – Binary - Age of the victim – Open-ended - Job position of the victim – Multiple choice - Frequency of previous experience of violence – Open-ended - Type of violence – Multiple choice - Status of reporting the incident – Binary - Reason for not reporting violence – Multiple choice - Severity of the violence – Unknown - Reasons of violence – Multiple choice - Typology of the assailant – Open-ended - Age of the assailant – Open-ended - Attitude of victim following the incident – Multiple choice - Suggestions to reduce future violence – Multiple choice |
| Colorado Hospital Association, 2022  Country: United States | - Study design: NA - Sample size: NA | - Name of reporting tool: Incident Review Form - No. of items: 14 - Approach of reporting: Physical form | - Date of the incident – Open-ended - Time of the incident – Open-ended - Location of the incident – Open-ended - Types of violence – Multiple choice and open-ended - Availability of witness – Binary and open-ended - Use of weapon – Binary - Injury status of victim – Binary - Severity of injury of the victim – Multiple choice - Description of the incident – Open-ended - Typology of assailant – Multiple choice and open-ended - Injury status of assailant – Binary - Severity of injury of the assailant – Multiple choice - Status of implementation of appropriate measures – Multiple choice and open-ended - Enforcement of law – Binary |
| Hamblin et al, 2017  Country: United States | - Study design: Randomised Controlled Trial - Sample size: 32 participants | - Name of reporting tool: Workplace Violence Form - No. of items: 6 - Approach of reporting: Online form | - Job position of the victim – Multiple choice - Description of the incident – Multiple choice - Types of violence – Multiple choice - Department where violence occurs – Multiple choice - Location of the incident – Multiple choice - Typology of assailant – Multiple choice |
| Health Professionals and Allied Employees, 2016  Country: United States | - Study design: NA - Sample size: NA | - Name of reporting tool: HPAE Workplace Violence Reporting Form - No. of items: 18 - Approach of reporting: Physical form | - Date of the incident – Open-ended - Time of the incident – Open-ended - Location of the incident – Open-ended - Report sent to hospital – Binary - Description of the incident – Multiple choice and open-ended - Injury status of the victim – Binary and open-ended - Name of the witness – Open-ended - Job title of the witness – Open-ended - Name of the supervisor – Open-ended - Date of reporting – Open-ended - Time of reporting – Open-ended - Name of the notifier – Open-ended - Typology of the assailant – Multiple choice - Disposition of the assailant – Multiple choice - Assailant’s risk factors – Multiple choice and open-ended - Action taken – Open-ended - Termination of the incident – Multiple choice - Use of restrain – Binary - Suggestion of preventive measure – Open-ended |
| Khedr et al, 2024  Country: Egypt | - Study design: Cross-Sectional Study - Sample size: 250 participants | - Name of reporting tool: NA - No. of items: 25 - Approach of reporting: Online form | **Personal and professional data**   - Age of the victim – Open-ended - Gender of the victim – Binary - Specialty of the victim – Multiple choice - Duration of working experience of the victim – Open-ended - Qualification of the victim – Multiple choice - Job position of the victim – Multiple choice   **Exposure to violence**   - Types of violence – Multiple choice   **Frequency and trend analysis of physical violence**   - Year(s) when physical violence occurred – Open-ended - Frequency of physical violence experienced per year – Open-ended - Relation between COVID-19 crisis and frequency of physical violence – Binary   **Analysis of the attacks of physical violence**   - Hospital where physical violence occurs – Multiple choice - Time of the incident – Multiple choice - Typology of the assailant – Multiple choice - Character of the assailant – Multiple choice - Reason for the of physical violence – Multiple choice - Instrument used in the physical violence – Multiple choice - Manner(s) of attack – Multiple choice - Part(s) of the body attacked during the physical violence – Multiple choice   **Consequences of physical violence**   - Immediate health effects of physical violence – Multiple choice - Long-term consequences of the violence – Multiple choice - Psychological effect of physical violence – Multiple choice - Reactions of physicians in the attacks of physical violence – Multiple choice - Legal outcome of the physical violence – Multiple choice   **Root causes of physical violence and proposed solutions**   - Root causes of increasing violence against physicians – Multiple choice - Proposed solutions for protecting physicians against violence in the future – Open-ended |
| Kim et al, 2023  Country: United States | - Study design: Cross-Sectional Study - Sample size: 96 participants | - Name of reporting tool: Violent Event Severity Tool (VEST) - No. of items: 14 - Approach of reporting: NA | - Age of the victim – Open-ended - Gender of the victim – Binary - Ethnicity of the victim– Multiple choice and open-ended - Qualification of the victim – Multiple choice and open-ended - Job position of the victim – Multiple choice and open-ended - Department of the victim – Multiple choice and open-ended - Duration of working experience of the victim – Open-ended - Previous experience of violence – Binary - Perceive that proactive identification of patients could prevent or reduce the severity of workplace violence – Binary - Perceive that automated notification could prevent or reduce the severity of workplace violence – Binary - Perceptions of safety at the workplace – Binary - Organisation has a violence prevention program – Binary - Types of violence – Multiple choice - Severity of violence – Multiple choice |
| Massachusetts Nurses Association, 2022  Country: United States | - Study design: NA - Sample size: NA | - Name of reporting tool: MNA Workplace Violence Reporting Form - No. of items: 19 - Approach of reporting: Physical form | - Name of the victim – Open-ended - Name of the hospital – Open-ended - Report sent to hospital – Binary - Date of the incident – Open-ended - Time of the incident – Open-ended - Location of the incident – Open-ended - Department of the incident – Open-ended - Description of the incident – Multiple choice and open-ended - Injury status of victim – Binary and open-ended - Presence of any witness – Binary - Typology of assailant – Multiple choice - Gender of assailant – Open-ended - Assailant’s risk factors – Multiple choice and open-ended - Disposition of the assailant – Multiple choice - Availability of assistance – Binary - Relevant personnel notified – Multiple choice - Take time off from work after the incident – Binary - Suggestion of preventive measure – Open-ended - Preference to be contacted for further assistance – Binary - Instrument (weapon) used in the violence – Binary and open-ended |
| McGuire et al, 2023  Country: United States | - Study design: Cross-Sectional Study - Sample size: 242 participants | - Name of reporting tool: NA - No. of items: 10 - Approach of reporting: Online form | - Gender of the victim – Binary - Job position of the victim – Multiple choice - Primary work shift of the victim – Multiple choice - Duration of working experience of the victim – Open-ended - Duration working in emergency department of the victim – Open-ended - Previous experience of verbal abuse in the prior six months – Binary - Previous experience of physical assault in the prior six months – Binary - Previous reporting of violence – Binary - Perceptions of safety at the workplace – Likert’ scale - Frequency of violence and reporting of violence in the prior six months – Likert’ scale |
| Ministry of Health Malaysia, 2022  Country: Malaysia | - Study design: NA - Sample size: NA | - Name of reporting tool: Violence Reporting Form 1 and 2 - No. of items: 37 - Approach of reporting: Physical form | - Name of the notifier – Open-ended - Job position of the notifier – Open-ended - Working address of the notifier – Open-ended - Contact number of the notifier – Open-ended - Name of the victim – Open-ended - Personal identification of the victim – Open-ended - Nationality of the victim – Open-ended - Gender of the victim – Open-ended - Ethnicity of the victim – Open-ended - Job position of the victim – Open-ended - Department of the victim – Open-ended - Duration of working experience – Open-ended - Name of the victim workplace – Open-ended - Working address of the victim workplace – Open-ended - Contact number of the victim – Open-ended - Date of the incident – Open-ended - Time of the incident – Open-ended - Location of the incident – Open-ended - List and contact of witness – Open-ended - Types of violence – Multiple choice and open-ended - Activities during the incident – Open-ended - Description of the incident – Open-ended - Injury status of the victim – Binary and open-ended - Typology of the assailant – Multiple choice and open-ended - Relationship of the assailant to the victim – Open-ended - Activation of alarm (Code Grey) – Binary and open-ended - Status of police report – Binary and open-ended - Treatment given to the victim – Binary and open-ended - Take time off from work after the incident – Open-ended - Debriefing given to the victim – Binary and open-ended - Mental health assessment to the victim after the incident – Open-ended - Referral of the victim to counsellor – Binary - Referral of the victim to psychiatry – Binary - Reasons of violence – Open-ended - Status of implementation of appropriate measures – Open-ended - Measures to prevent future violence – Open-ended |
| Odes et al, 2022  Country: United States | - Study design: Cross-Sectional Study - Sample size: 413 facilities | - Name of reporting tool: Workplace Violent Incident Reporting System (WVIRS) - No. of items: 20 - Approach of reporting: Online form | - Name of the hospital – Open-ended - Date of the incident – Open-ended - Time of incident – Multiple choice - Location of the incident – Multiple choice - Types of violence – Multiple choice - Number of staffs injured – Multiple choice - Types of injury sustained by victim – Multiple choice - Activities during the incident – Multiple choice - Incident occurs when the victim was alone with the assailant – Binary - Incident occurs when the victim was in an isolated area – Binary - Incident occurs in a location that was unfamiliar or new to the victim – Binary - Incident occurs when the victim is performing an unfamiliar or new task – Multiple choice - Other non-hospital staff (contractor) affected by the incident – Binary and open-ended - Personnel who assisted the victim during the incident – Multiple choice - Typology of assailant – Multiple choice - Reporting of the incident to the nearest OSHA district office – Binary - The name of OSHA district office in which incident is reported to – Open-ended - Assistance provided by the local law enforcement – Multiple choice - Continuing threats to the victim due to unresolved control measures – Binary - Measures to prevent future violence – Multiple choice |
| Olabisi et al, 2025  Country: Nigeria | - Study design: Mixed-Methods Study - Sample size: 401 participants | - Name of reporting tool: Workplace violence questionnaire (WVQ) - No. of items: 36 - Approach of reporting: Physical form | **Sociodemographic information**   - Age of the victim – Open-ended - Gender of the victim – Binary - Marital status of the victim – Multiple choice - Highest academic qualification of the victim – Multiple choice - Professional qualifications of the victim – Multiple choice and open-ended - Religion of the victim – Binary - Job position of the victim – Binary - Job title of the victim – Multiple choice and open-ended - Duration of working experience of the victim – Open-ended - Level of healthcare of the victim (primary, secondary, tertiary) – Multiple choice - Department of the victim – Open-ended - Work in shifts – Binary - Gender of patients whom victim most frequently works with – Multiple choice   **Pattern of workplace violence**   - Types of violence experienced in the past 12 months – Multiple choice - Frequency of experiencing violence in the last 12 months – Likert’s scale - Time of the incident – Multiple choice - Day of the week when the violence occurs – Multiple choice - Typology of the assailant – Multiple choice   **Protocol for prevention of workplace violence**   - Level of worry about violence in the current workplace – Likert’s scale - Presence of reporting procedure of violence in the workplace – Binary - Presence of encouragement to report violence in the workplace - Binary - Organisation has a violence prevention program – Multiple choice - Measures to prevent future violence – Multiple choice - Perception that these measures would be helpful in preventing violence in the workplace – Likert’s scale   **Protocol for managing workplace violence**   - Action taken – Multiple choice - Perception that the violence could have been prevented – Multiple choice - Injury status of the victim – Multiple choice - Victim needs formal treatment for the injuries resulting from violence – Multiple choice - Take time off from work after the incident – Multiple choice - Number of days victim take time from work due to the violence – Multiple choice - Action taken to investigate the cause of the violence – Multiple choice - Personnel who took the action – Multiple choice - Consequence to the assailant – Multiple choice - Assistance provided by employer or supervisor – Multiple choice - Level of satisfaction of the victim with the manner in which the violence was handle – Likert’s scale - Reason of not reporting violence – Multiple choice |
| Pompeii et al, 2016  Country: United States | - Study design: Mixed-Methods Study - Sample size: 6 hospitals, 11,000 participants | - Name of reporting tool: NA - No. of items: 16 - Approach of reporting: Physical and online form | - Gender of the victim – Binary - Age of the victim – Open-ended - Ethnicities of the victim – Multiple choice - Duration of working experience in hospital – Open-ended - Job position of the victim – Multiple choice and open-ended - Previous experiences with violence in the prior year – Binary - Types of the violence – Multiple choice - Victim frightened or worry about personal safety – Binary - Injury status of the victim – Binary - Use of weapon during the violence – Binary - Perceived intent to harm – Binary - Victim was alone during the violence – Binary - Typology of the assailant – Binary - Consequences of the violence to the victim – Open-ended - Personnel to whom the violence was reported – Open-ended - Reason of not reporting violence – Multiple choice and open-ended |
| Ramacciati et al, 2021  Country: Italy | - Study design: Cross-Sectional Study - Sample size: 184 participants | - Name of reporting tool: PSaggress app - No. of items: 5 - Approach of reporting: Mobile application | - Date of the incident – Open-ended - Time of the incident – Open-ended - Types of violence – Multiple choice - Typology of assailant – Multiple choice - Reason of violence – Open-ended |
| Renwick et al, 2016  Country: United Kingdom | - Study design: Secondary data analysis - Sample size: 552 incidents | - Name of reporting tool: Reporting of Injuries, Diseases and Dangerous Occurrences Regulations Form - No. of items: 28 - Approach of reporting: Online form | - Name of the victim – Open-ended - Gender of the victim – Open-ended - Age of the victim – Open-ended - Job position of the victim – Multiple choice - Employment status of the victim – Multiple choice and open-ended - Address of the hospital – Open-ended - Date of the incident – Open-ended - Time of the incident – Open-ended - Types of injury sustained by the victim – Multiple choice - Department which the incident occurs – Open-ended - Local authority under which the incident occurs – Multiple choice - Types of industry which the incident occurs – Multiple choice - Main activity at the workplace – Multiple choice - Types of the violence – Multiple choice and open-ended - Reason of violence – Multiple choice and open-ended - Activities during the incident – Open-ended - Activities lead to the incident – Open-ended - Environmental conditions at the time of the incident – Open-ended - Name and type of the equipment or substances involved in the incident – Open-ended - Other relevant information which describes the incident – Open-ended - Victim deceased as a result of the injury – Binary - Victim sustained injury in hospital premises – Binary - Victim treated in hospital as a result of the incident – Binary - Victim sustained a specified injury – Binary - Nature of the injuries sustained – Open-ended - Body part injured – Multiple choice - The incident prevents the victim from carrying out their routine work for more than 7 days – Binary - Measures to prevent future violence – Open-ended |
| Richardson et al, 2018  Country: New Zealand | - Study design: Cross-sectional Study - Sample size: 107 participants | - Name of reporting tool: NA - No. of items: 7 - Approach of reporting: Physical form | - Job position of the victim – Multiple choice - Gender of the victim – Binary - Duration of incident from the commencement of shift – Open-ended - Day (of the week) of the incident – Multiple choice - Time of the incident – Multiple choice - Location of the incident – Multiple choice - Description of the incident – Multiple choice and open-ended |
| WHO, 2003  Country: Multiple | - Study design: Mixed-methods study - Sample size: 6099 participants | - Name of reporting tool: Workplace Violence in the Health Sector Country Case Studies Research Questionnaire - No. of items: 114 - Approach of reporting: Physical form | **Personal and Workplace Data**   - Age of the victim – Multiple choice - Gender of the victim – Binary - Marital status of the victim – Multiple choice - Migration status of the victim – Binary - Date of arrival in the country – Multiple choice - Victim’s perceived ethnic majority or minority – Binary - Job position of the victim – Multiple choice and open-ended - Job category of the victim – Multiple choice and open-ended - Duration of working experience – Multiple choice - Employment sector of victim – Multiple choice and open-ended - Victim’s mode of working – Multiple choice - Victim’s status of shift work – Binary - Status of night shift – Binary - Victim’s interaction with patients at work – Binary - Victim’s routine direct physical contact with patients – Binary - Types of patients most frequently work with – Binary - Gender of patients most frequently work with – Multiple choice - Specialty of the victim – Multiple choice - Location where victim spend most of the time – Multiple choice and open-ended - No. of staff present in the same work setting with victim – Multiple choice - Victim’s level of worry about violence in the workplace – Likert’s scale - Availability of violence reporting procedure in the workplace – Binary - Knowing how to use the reporting tool – Binary - Availability of encouragement to report workplace violence – Binary - Personnel who encourage the reporting of violence– Multiple choice and open-ended   **Physical Violence**   - History of physical violence in the past one year – Binary - Use of weapon during the last physical violence – Binary - Perception that physical violence is typical in the workplace – Binary - Typology of assailant – Multiple choice and open-ended - Location of the incident – Multiple choice - Time of the incident – Multiple choice - Day of the incident – Multiple choice - Response towards the incident – Multiple choice and open-ended - Perception that the previous incident could be prevented – Binary - Status of injury in the previous incident – Binary - Receive of treatment after the incident – Binary - Attitude after the incident – Likert’s scale - Take time off from work after the incident – Binary - Duration of time off after the incident – Multiple choice - Action taken to investigate the causes of the incident – Multiple choice - Personnel who took the action – Multiple choice and open-ended - Consequences to the assailant – Multiple choice and open-ended - Provision of support from employer – Binary - Level of satisfaction with the handling of the incident – Likert’s scale - Reasons of not reporting violence – Multiple choice and open-ended - Witness of physical violence – Binary - Frequency of witnessing physical violence – Multiple choice - Reporting of workplace violence in the past one year – Binary - Disciplinary action taken for reporting workplace violence – Binary   **Verbal Abuse**   - History of verbal abuse in the past one year – Binary - Frequency of verbal abuse in the past one year – Multiple choice - Typology of assailant in the last abuse – Multiple choice and open-ended - Perception that vocal abuse is typical in the workplace – Binary - Location of the incident – Multiple choice - Response towards the incident – Multiple choice and open-ended - Attitude after the incident – Likert’s scale - Perception that the abuse could be prevented – Binary - Action taken to investigate the causes of the incident – Multiple choice - Personnel who took action – Multiple choice and open-ended - Consequences to the assailant – Multiple choice and open-ended - Provision of support from employer – Binary - Level of satisfaction with the handling of the incident – Likert’s scale - Reasons of not reporting violence – Multiple choice and open-ended   **Bullying or Mobbing**   - History of bullying or mobbing in the past one year – Binary - Frequency of bullying or mobbing in the past one year – Multiple choice - Typology of assailant in the last bullying or mobbing – Multiple choice and open-ended - Perception that bullying or mobbing is typical in the workplace – Binary - Location of the incident – Multiple choice - Response towards the incident – Multiple choice and open-ended - Attitude after the incident – Likert’s scale - Perception that the bullying or mobbing can be prevented – Binary - Action taken to investigate the causes of the incident – Multiple choice - Personnel who took action – Multiple choice and open-ended - Consequences to the assailant – Multiple choice and open-ended - Provision of support from employer – Binary - Level of satisfaction with the handling of the incident – Likert’s scale - Reasons of not reporting violence – Multiple choice and open-ended   **Sexual Harassment**   - History of sexual harassment in the past one year – Binary - Frequency of sexual harassment in the past one year – Multiple choice - Typology of assailant in the last sexual harassment – Multiple choice and open-ended - Perception that sexual harassment is typical in the workplace – Binary - Location of the incident – Multiple choice - Response towards the incident – Multiple choice and open-ended - Attitude after the incident – Likert’s scale - Perception that the abuse can be prevented – Binary - Action taken to investigate the causes of the incident – Multiple choice - Personnel who took action – Multiple choice and open-ended - Consequences to the assailant – Multiple choice and open-ended - Provision of support from employer – Binary - Level of satisfaction with the handling of the incident – Likert’s scale - Reasons of not reporting violence – Multiple choice and open-ended   **Racial Harassment**   - History of racial harassment in the past one year – Binary - Frequency of racial harassment in the past one year – Multiple choice - Typology of assailant in the last racial harassment – Multiple choice and open-ended - Perception that racial harassment is typical in the workplace – Binary - Location of the incident – Multiple choice - Response towards the incident – Multiple choice and open-ended - Attitude after the incident – Likert’s scale - Perception that the abuse can be prevented – Binary - Action taken to investigate the causes of the incident – Multiple choice - Personnel who took action – Multiple choice and open-ended - Consequences to the assailant – Multiple choice and open-ended - Provision of support from employer – Binary - Level of satisfaction with the handling of the incident – Likert’s scale - Reasons of not reporting violence – Multiple choice and open-ended   **Health Sector Employer**   - Presence of violence policies at workplace – Multiple choice - Availability of measures to deal with workplace violence at the workplace – Multiple choice and open-ended - Perception that measures would be helpful in the workplace – Likert’s scale - Implementation of changes in the workplace in the past two years – Multiple choice and open-ended - Impact of the changes on daily work – Multiple choice and open-ended   **Opinions on Workplace Violence**   - Risk factors of physical violence at workplace – Open-ended - Risk factors of non-physical violence at workplace – Open-ended - Suggestions of measures to reduce violence at the workplace – Open-ended |
