# Supplementary material for: How to Adequately Report Workplace Violence in Healthcare Setting: A Systematic Review With Hierarchical Cluster Analysis of Workplace Violence Reporting Forms
Source: J Nurs Manag. 2026 Jun 28;2026:4803748. doi: 10.1155/jonm/4803748 (PMC13310381; doi:10.1155/jonm/4803748)
Supplement: Supplementary file 3 — Supporting Information 3 Supporting File 3: Detailed domains and items of each WPV reporting form. [file JONM-2026-4803748-s004.docx]

| **Domains** | **Items** | **Arnetz, 1998** | **Arnetz, 2011** | **Bowers et al, 2005** | **Byon et al, 2022** | **Calik et al, 2021** | **California Hospital Association, 2019** | **Cikriklar et al, 2016** | **Colorado Hospital Association, 2022** | **Hamblin et al, 2017** | **Health Professionals and Allied Employees, 2016** | **Khedr et al, 2024** | **Kim et al, 2023** | **Massachusetts Nurses Association, 2022** | **McGuire et al, 2023** | **Ministry of Health Malaysia, 2022** | **Odes et al, 2022** | **Olabisi et al, 2025** | **Pompeii et al, 2016** | **Ramacciati et al, 2021** | **Renwick et al, 2016** | **Richardson et al, 2018** | **WHO, 2003** | **Total** |
| --- | --- | --- | --- | --- | --- | --- | --- | --- | --- | --- | --- | --- | --- | --- | --- | --- | --- | --- | --- | --- | --- | --- | --- | --- |
| Sociodemographic Data of the Victim | Name of the victim | O |  |  |  |  | O |  |  |  |  |  |  | O |  | O |  |  |  |  | O |  |  | 5 |
|  | Identification card number of the victim |  |  |  |  |  |  |  |  |  |  |  |  |  |  | O |  |  |  |  |  |  |  | 1 |
|  | Contact number of the victim |  |  |  |  |  | O |  |  |  |  |  |  |  |  | O |  |  |  |  |  |  |  | 2 |
|  | Gender of the victim | M | B |  | B | B |  | B |  |  |  | B | B |  | B | O |  | B | B |  | O | B | B | 14 |
|  | Age of the victim | M | O |  | O | O |  | O |  |  |  | O | O |  |  |  |  | O | O |  | O |  | M | 11 |
|  | Nationality of the victim |  |  |  |  |  |  |  |  |  |  |  |  |  |  | O |  |  |  |  |  |  | B | 2 |
|  | Date of arrival in the country of the victim (for foreigner) |  |  |  |  |  |  |  |  |  |  |  |  |  |  |  |  |  |  |  |  |  | M | 1 |
|  | Ethnicity of the victim |  |  |  | M |  |  |  |  |  |  |  | MO |  |  | O |  |  | M |  |  |  | B | 5 |
|  | Marital status of the victim |  |  |  |  |  |  |  |  |  |  |  |  |  |  |  |  | M |  |  |  |  | B | 2 |
|  | Working address of the victim |  |  |  |  |  |  |  |  |  |  |  |  |  |  | O |  |  |  |  |  |  |  | 1 |
|  | Religion of the victim |  |  |  |  |  |  |  |  |  |  |  |  |  |  |  |  | B |  |  |  |  |  | 1 |
| Job Characteristics of the Victim | Job position of the victim | MO | M |  |  | M | O | M |  | M |  | M | MO |  | M | O |  | B | MO |  | M | M | MO | 15 |
|  | Job title of the victim |  |  |  |  |  |  |  |  |  |  |  |  |  |  |  |  | MO |  |  |  |  | MO | 2 |
|  | Department (or specialty) of the victim |  |  |  | M |  |  |  |  |  |  |  | MO |  |  | O |  | O |  |  |  |  | M | 5 |
|  | Victim’s mode of working (shift) |  |  |  |  |  |  |  |  |  |  |  |  |  |  |  |  |  |  |  |  |  | M | 1 |
|  | Primary working shift of the victim |  |  |  |  |  |  |  |  |  |  |  |  |  | M |  |  |  |  |  |  |  |  | 1 |
|  | Work in shift |  |  |  |  |  |  |  |  |  |  |  |  |  |  |  |  | B |  |  |  |  | B | 2 |
|  | Number of workers on duty in every shift |  |  | O |  |  |  |  |  |  |  |  |  |  |  |  |  |  |  |  |  |  | M | 2 |
|  | Number of incidents in every shift |  |  | O |  |  |  |  |  |  |  |  |  |  |  |  |  |  |  |  |  |  |  | 1 |
|  | Number of containments in every shift |  |  | O |  |  |  |  |  |  |  |  |  |  |  |  |  |  |  |  |  |  |  | 1 |
|  | Qualification of the victim |  |  |  |  |  |  |  |  |  |  | M | MO |  |  |  |  | MO |  |  |  |  |  | 3 |
|  | Specialty of the victim |  |  |  |  |  |  |  |  |  |  | M |  |  |  |  |  |  |  |  |  |  |  | 1 |
|  | Place of employment of the victim | O | M |  |  |  |  |  |  |  |  |  |  |  |  |  |  |  |  |  |  |  |  | 2 |
|  | Employment status of the victim |  | M |  |  |  |  |  |  |  |  |  |  |  |  |  |  |  |  |  | MO |  |  | 2 |
|  | Name of the hospital |  |  |  |  |  |  |  |  |  |  |  |  | O |  |  |  |  |  |  |  |  |  | 1 |
|  | Regions of hospital |  |  |  | M |  |  |  |  |  |  |  |  |  |  |  |  |  |  |  |  |  |  | 1 |
|  | Level of healthcare (primary, secondary, tertiary) |  |  |  |  |  |  |  |  |  |  |  |  |  |  |  |  | M |  |  |  |  | MO | 2 |
|  | Supervisor status |  | M |  |  |  |  |  |  |  |  |  |  |  |  |  |  |  |  |  |  |  |  | 1 |
|  | Name of the supervisor |  |  |  |  |  |  |  |  |  | O |  |  |  |  |  |  |  |  |  |  |  |  | 1 |
|  | Duration of working experience in healthcare of the victim |  | O |  |  |  |  |  |  |  |  | O | O |  | O | O |  | O | O |  |  |  | M | 8 |
|  | Duration of working experience in the hospital of the victim |  | O |  | O |  |  |  |  |  |  |  |  |  |  |  |  |  |  |  |  |  |  | 2 |
|  | Duration working in emergency department of the victim |  |  |  |  |  |  |  |  |  |  |  |  |  | O |  |  |  |  |  |  |  |  | 1 |
|  | Previous experience (frequency) of violence |  | M |  | M |  |  | O |  |  |  | O | B |  | B |  |  | L | B |  |  |  | B | 9 |
|  | Paid productive hours (PPH) of the victim |  | X |  |  |  |  |  |  |  |  |  |  |  |  |  |  |  |  |  |  |  |  | 1 |
|  | Victim’s routine direct physical contact with patients |  |  |  |  |  |  |  |  |  |  |  |  |  |  |  |  |  |  |  |  |  | B | 1 |
|  | Types of patients most frequently work with |  |  |  |  |  |  |  |  |  |  |  |  |  |  |  |  |  |  |  |  |  | B | 1 |
|  | No. of staff present in the same work setting with victim |  |  |  |  |  |  |  |  |  |  |  |  |  |  |  |  |  |  |  |  |  | M | 1 |
|  | Location where victim spend most of the time |  |  |  |  |  |  |  |  |  |  |  |  |  |  |  |  |  |  |  |  |  | MO | 1 |
|  | Experience more violence than before COVID-19 |  |  |  |  |  |  |  |  |  |  | B |  |  |  |  |  |  |  |  |  |  |  | 1 |
|  | Gender of patients whom victim most frequently works with |  |  |  |  |  |  |  |  |  |  |  |  |  |  |  |  | M |  |  |  |  | M | 2 |
| Characteristics of the Notifier | Name of the notifier |  |  |  |  |  |  |  |  |  | O |  |  |  |  | O |  |  |  |  |  |  |  | 2 |
|  | Job position of the notifier |  |  |  |  |  |  |  |  |  |  |  |  |  |  | O |  |  |  |  |  |  |  | 1 |
|  | Work address of the notifier |  |  |  |  |  |  |  |  |  |  |  |  |  |  | O |  |  |  |  |  |  |  | 1 |
|  | Contact number of the notifier |  |  |  |  |  |  |  |  |  |  |  |  |  |  | O |  |  |  |  |  |  |  | 1 |
| Characteristics of the Incident | Date of the incident | O | O |  |  |  | O |  | O |  | O |  |  | O |  | O | O |  |  | O |  |  |  | 9 |
|  | Year of the incident |  |  |  |  | O |  |  |  |  |  | O |  |  |  |  |  |  |  |  | O |  |  | 3 |
|  | Season of the incident |  |  |  |  | O |  |  |  |  |  |  |  |  |  |  |  |  |  |  |  |  |  | 1 |
|  | Month of the incident |  |  |  |  | O |  |  |  |  |  |  |  |  |  |  |  |  |  |  |  |  |  | 1 |
|  | Day (of the week) when the incident occurs |  |  |  |  |  |  |  |  |  |  |  |  |  |  |  |  | M |  |  |  | M | M | 3 |
|  | Time of the incident | O | O |  |  | O | O |  | O |  | O | M |  | O |  | O | M | M |  | O | O | M | M | 15 |
|  | Work shift in which the incident occurs |  | X |  |  |  |  |  |  |  |  |  |  |  |  |  |  |  |  |  |  |  |  | 1 |
|  | Local authority under which the incident occurs |  |  |  |  |  |  |  |  |  |  |  |  |  |  |  |  |  |  |  | M |  |  | 1 |
|  | Types of industry which the incident occurs |  |  |  |  |  |  |  |  |  |  |  |  |  |  |  |  |  |  |  | M |  |  | 1 |
|  | Hospital where the incident occurs |  |  |  |  |  |  |  |  |  |  | M |  |  |  | O | O |  |  |  |  |  |  | 3 |
|  | Address of the hospital |  |  |  |  |  |  |  |  |  |  |  |  |  |  | O |  |  |  |  | O |  |  | 2 |
|  | Location (Department) of the incident | MO | M |  |  | M | O |  | O | M | O |  |  | O |  | O | M |  |  |  | O | M | M | 13 |
|  | Instrument (weapon) used in the violence |  | O |  |  |  |  |  | B |  |  | M |  | BO |  |  |  |  | B |  | O |  | B | 7 |
|  | Main activity at the workplace |  |  |  |  |  |  |  |  |  |  |  |  |  |  |  |  |  |  |  | M |  |  | 1 |
|  | Types of violence | MO | M |  |  | O |  | M | MO | M |  | M | M |  |  | MO | M | M | M | M | MO |  |  | 14 |
|  | Description of the incident |  |  |  |  |  | O |  | O | M | MO |  |  | MO |  | O |  |  |  |  | O | MO |  | 8 |
|  | Reason(s) of violence |  |  |  |  | M |  | M |  |  |  | M |  |  |  | O |  |  |  | O | MO |  |  | 6 |
|  | Severity of the violence |  |  |  |  |  |  | X |  |  |  |  | M |  |  |  |  |  |  |  |  |  |  | 2 |
|  | Manner(s) of attack |  |  |  |  |  |  |  |  |  |  | M |  |  |  |  |  |  |  |  |  |  |  | 1 |
|  | Activities preceding or leading to the incident | MO | O |  |  |  |  |  |  |  |  |  |  |  |  |  |  |  |  |  | O |  |  | 3 |
|  | Activities during the incident | MO |  |  |  |  |  |  |  |  |  |  |  |  |  | O | M |  |  |  | O |  |  | 4 |
|  | Environmental conditions at the time of the incident |  |  |  |  |  |  |  |  |  |  |  |  |  |  |  |  |  |  |  | O |  |  | 1 |
|  | Feeling of the victim in advance | M |  |  |  |  |  |  |  |  |  |  |  |  |  |  |  |  |  |  |  |  |  | 1 |
|  | Victim was alone or in an isolated area | M |  |  |  |  |  |  |  |  |  |  |  |  |  |  | B |  | B |  |  |  |  | 3 |
|  | Victim work in a location that was unfamiliar or new |  |  |  |  |  |  |  |  |  |  |  |  |  |  |  | B |  |  |  |  |  |  | 1 |
|  | Victim is performing an unfamiliar or new task |  |  |  |  |  |  |  |  |  |  |  |  |  |  |  | M |  |  |  |  |  |  | 1 |
|  | Duration of incident from the commencement of shift | M |  |  |  |  |  |  |  |  |  |  |  |  |  |  |  |  |  |  |  | O |  | 2 |
|  | Other non-hospital staff affected |  |  |  |  |  |  |  |  |  |  |  |  |  |  |  | BO |  |  |  |  |  |  | 1 |
|  | Continuing threats to the victim |  |  |  |  |  |  |  |  |  |  |  |  |  |  |  | B |  |  |  |  |  |  | 1 |
| Characteristics of the Injury | Part(s) of the body attacked or injured |  |  |  |  |  |  |  |  |  |  | M |  |  |  |  |  |  |  |  | M |  |  | 2 |
|  | Injury status of the victim |  | M |  |  |  |  |  | B |  | BO |  |  | BO |  | BO |  | M | B |  | B |  | B | 9 |
|  | Types of injury sustained by the victim |  |  |  |  |  |  |  |  |  |  |  |  |  |  |  | M |  |  |  | M |  |  | 2 |
|  | Severity of injury of the victim |  |  |  |  |  |  |  | M |  |  |  |  |  |  |  |  |  |  |  | O |  |  | 2 |
|  | Victim deceased as a result of the injury |  |  |  |  |  |  |  |  |  |  |  |  |  |  |  |  |  |  |  | B |  |  | 1 |
|  | Injury status of the assailant |  |  |  |  |  |  |  | B |  |  |  |  |  |  |  |  |  |  |  |  |  |  | 1 |
|  | Severity of injury of the assailant |  |  |  |  |  |  |  | M |  |  |  |  |  |  |  |  |  |  |  |  |  |  | 1 |
|  | Number of staffs injured |  |  |  |  |  |  |  |  |  |  |  |  |  |  |  | M |  |  |  |  |  |  | 1 |
| Measures Taken During the Violence | Action (or reaction) taken | M |  |  |  |  |  |  |  |  | O | M |  |  |  |  |  | M |  |  |  |  | MO | 5 |
|  | Personnel who took the action or provide assistance |  |  |  |  |  |  |  |  |  |  |  |  |  |  |  | M | M |  |  |  |  | MO | 3 |
|  | Risk reduction measures towards the assailant |  |  |  |  |  | O |  |  |  |  |  |  |  |  |  |  |  |  |  |  |  |  | 1 |
|  | Status of implementation of appropriate measures |  |  |  |  |  | BO |  | MO |  |  |  |  |  |  | O |  |  |  |  |  |  |  | 3 |
|  | Status of availability of alarm (or other assistance) during the incident |  |  |  |  |  | O |  |  |  |  |  |  |  |  |  |  |  |  |  |  |  |  | 1 |
|  | Use alarm (or other assistance) during the incident |  |  |  |  |  | O |  |  |  |  |  |  |  |  | BO |  |  |  |  |  |  |  | 2 |
|  | Response of staff or law enforcer |  |  |  |  |  | O |  |  |  |  |  |  |  |  |  |  |  |  |  |  |  |  | 1 |
|  | Enforcement of law or legal outcome |  |  |  |  |  |  |  | B |  |  | M |  |  |  |  | M |  |  |  |  |  |  | 3 |
|  | Status of police report | M |  |  |  |  |  |  |  |  |  |  |  |  |  | BO |  |  |  |  |  |  |  | 2 |
|  | Termination of the incident |  |  |  |  |  |  |  |  |  | M |  |  |  |  |  |  |  |  |  |  |  |  | 1 |
|  | Use of restrain |  |  |  |  |  |  |  |  |  | B |  |  |  |  |  |  |  |  |  |  |  |  | 1 |
|  | Assistance or support provided by employer or supervisor |  |  |  |  |  |  |  |  |  |  |  |  | B |  |  |  | M |  |  |  |  | B | 3 |
|  | Organisation has a violence prevention program |  |  |  |  |  |  |  |  |  |  |  | B |  |  |  |  | M |  |  |  |  | MO | 3 |
|  | Presence of violence policies at workplace |  |  |  |  |  |  |  |  |  |  |  |  |  |  |  |  |  |  |  |  |  | M | 1 |
| Consequences of the Violence | Immediate health effects (consequence) of the violence to the victim | MO |  |  |  |  |  |  |  |  |  | M |  |  |  |  |  |  | O |  |  |  |  | 4 |
|  | Long-term consequences of the violence to the victim |  |  |  |  |  |  |  |  |  |  | M |  |  |  |  |  |  |  |  |  |  |  | 1 |
|  | Time lost due to the violence |  | B |  |  |  |  |  |  |  |  |  |  |  |  |  |  |  |  |  |  |  |  | 1 |
|  | Take time off from work after the incident |  |  |  |  |  |  |  |  |  |  |  |  | B |  | O |  | M |  |  | B |  | B | 5 |
|  | Number of days victim take time from work |  |  |  |  |  |  |  |  |  |  |  |  |  |  |  |  | M |  |  |  |  | M | 2 |
|  | Consequences to the assailant |  |  |  |  |  |  |  |  |  |  |  |  |  |  |  | B | M |  |  |  |  | MO | 3 |
| Post-Violence Treatment | Treatment given to the victim |  |  |  |  |  |  |  |  |  |  |  |  |  |  | BO |  | M |  |  | B |  | B | 4 |
|  | Debriefing given to the victim |  |  |  |  |  |  |  |  |  |  |  |  |  |  | BO |  |  |  |  |  |  |  | 1 |
|  | Mental health assessment to the victim after the incident |  |  |  |  |  |  |  |  |  |  |  |  |  |  | O |  |  |  |  |  |  |  | 1 |
|  | Referral of the victim to counsellor |  |  |  |  |  |  |  |  |  |  |  |  |  |  | B |  |  |  |  |  |  |  | 1 |
|  | Referral of the victim to psychiatry |  |  |  |  |  |  |  |  |  |  |  |  |  |  | B |  |  |  |  |  |  |  | 1 |
| Aftermath of the Violence | Measures taken to prevent future violence |  |  |  |  |  |  |  |  |  |  |  |  |  |  | O |  | M |  |  | O |  |  | 3 |
|  | Action taken to investigate the cause of the violence |  |  |  |  |  |  |  |  |  |  |  |  |  |  |  |  | M |  |  |  |  | M | 2 |
|  | Suggestion of measures to prevent future violence |  |  |  |  |  | O | M |  |  | O | O |  | O |  | O | M | M |  |  |  |  | O | 9 |
|  | Implementation of changes in the workplace |  |  |  |  |  |  |  |  |  |  |  |  |  |  |  |  |  |  |  |  |  | MO | 1 |
|  | Impact of the changes on daily work |  |  |  |  |  |  |  |  |  |  |  |  |  |  |  |  |  |  |  |  |  | MO | 1 |
| Reporting of the Violence | Status of reporting the incident |  |  |  |  |  |  | B |  |  | B |  |  | B |  |  | B |  |  |  |  |  | B | 5 |
|  | Personnel to whom the violence was reported |  |  |  |  |  |  |  |  |  |  |  |  |  |  |  |  |  | O |  |  |  |  | 1 |
|  | Reporting date | O |  |  |  |  | O |  |  |  | O |  |  |  |  |  |  |  |  |  |  |  |  | 3 |
|  | Reporting time |  |  |  |  |  | O |  |  |  | O |  |  |  |  |  |  |  |  |  |  |  |  | 2 |
|  | Presence of reporting procedure of violence in the workplace |  |  |  |  |  |  |  |  |  |  |  |  |  |  |  |  | B |  |  |  |  | B | 2 |
|  | Familiarity of reporting |  | B |  |  |  |  |  |  |  |  |  |  |  |  |  |  |  |  |  |  |  | B | 2 |
|  | Previous reporting of violence |  | MO |  |  |  |  |  |  |  |  |  |  |  | B |  |  |  |  |  |  |  |  | 2 |
|  | Frequency of reporting violence |  |  |  |  |  |  |  |  |  |  |  |  |  | L |  |  |  |  |  |  |  |  | 1 |
|  | Reporting of violence using another approach |  | MO |  |  |  |  |  |  |  |  |  |  | B |  |  |  |  |  |  |  |  |  | 2 |
|  | Reason for not reporting violence |  |  |  |  |  |  | M |  |  |  |  |  |  |  |  |  | M | MO |  |  |  | MO | 4 |
|  | Presence of encouragement to report violence in the workplace |  |  |  |  |  |  |  |  |  |  |  |  |  |  |  |  |  |  |  |  |  | B | 1 |
|  | Personnel who encourage the reporting of violence |  |  |  |  |  |  |  |  |  |  |  |  |  |  |  |  |  |  |  |  |  | MO | 1 |
|  | Disciplinary action taken for reporting workplace violence |  |  |  |  |  |  |  |  |  |  |  |  |  |  |  |  |  |  |  |  |  | B | 1 |
|  | Difficulty of reporting violence than before COVID-19 |  |  |  | M |  |  |  |  |  |  |  |  |  |  |  |  |  |  |  |  |  |  | 1 |
| Perception of Victim Post-Violence | Perceived cause of the increasing violence |  |  |  |  |  | O |  |  |  |  | M |  |  |  |  |  |  |  |  |  |  |  | 2 |
|  | Perception that the violence could have been prevented |  |  |  |  |  |  |  |  |  |  |  | B |  |  |  |  | M |  |  |  |  | B | 3 |
|  | Perception that violence is typical in the workplace |  |  |  |  |  |  |  |  |  |  |  |  |  |  |  |  |  |  |  |  |  | B | 1 |
|  | Perception that preventive measures would be helpful |  |  |  |  |  |  |  |  |  |  |  |  |  |  |  |  | L |  |  |  |  | L | 2 |
|  | Attitude of victim following the incident |  |  |  |  |  |  | M |  |  |  |  |  |  |  |  |  |  |  |  |  |  | L | 2 |
|  | Perceptions of safety at the workplace |  |  |  |  |  |  |  |  |  |  |  | B |  | L |  |  |  | B |  |  |  |  | 3 |
|  | Preference to be contacted for further assistance |  |  |  |  |  |  |  |  |  |  |  |  | B |  |  |  |  |  |  |  |  |  | 1 |
|  | Level of worry about violence |  |  |  |  |  |  |  |  |  |  |  |  |  |  |  |  | L |  |  |  |  | L | 2 |
|  | Level of satisfaction of the victim with the manner in which the violence was handle |  |  |  |  |  |  |  |  |  |  |  |  |  |  |  |  | L |  |  |  |  | L | 2 |
|  | Perceived intent to harm |  |  |  |  |  |  |  |  |  |  |  |  |  |  |  |  |  | B |  |  |  |  | 1 |
| Characteristics of the Assailant | Age of the assailant | M |  |  |  |  |  | O |  |  |  |  |  |  |  |  |  |  |  |  |  |  |  | 2 |
|  | Gender of the assailant | M |  |  |  |  |  |  |  |  |  |  |  | O |  |  |  |  |  |  |  |  |  | 2 |
|  | Assailant’s patient registration number | O |  |  |  |  |  |  |  |  |  |  |  |  |  |  |  |  |  |  |  |  |  | 1 |
|  | Typology of the assailant | MO |  |  |  | M |  | O | MO | M | M | M |  | M |  | MO | M | M | B | M |  |  | MO | 14 |
|  | Conditions or characters of the assailant | M |  |  |  |  |  |  |  |  |  | M |  |  |  |  |  |  |  |  |  |  |  | 2 |
|  | Assailant’s risk factors |  |  |  |  |  | O |  |  |  | MO |  |  | MO |  |  |  |  |  |  |  |  | O | 4 |
|  | Assailant’s history of violence |  |  |  |  |  | O |  |  |  |  |  |  |  |  |  |  |  |  |  |  |  |  | 1 |
|  | Disposition of the assailant |  |  |  |  |  |  |  |  |  | M |  |  | M |  |  |  |  |  |  |  |  |  | 2 |
|  | Relationship of the assailant to the victim |  |  |  |  |  |  |  |  |  |  |  |  |  |  | O |  |  |  |  |  |  |  | 1 |
| Characteristics of the Witness | Availability of witness |  |  |  |  |  |  |  | BO |  |  |  |  | B |  |  |  |  |  |  |  |  | B | 3 |
|  | Name of the witness |  | O |  |  |  |  |  |  |  | O |  |  |  |  | O |  |  |  |  |  |  |  | 3 |
|  | Job title of the witness |  |  |  |  |  |  |  |  |  | O |  |  |  |  |  |  |  |  |  |  |  |  | 1 |
|  | Contact of witness |  |  |  |  |  |  |  |  |  |  |  |  |  |  | O |  |  |  |  |  |  |  | 1 |
|  | Frequency of witnessing physical violence |  |  |  |  |  |  |  |  |  |  |  |  |  |  |  |  |  |  |  |  |  | M | 1 |

Blue: Cluster 1 (rapid WPV reporting form); Green: Cluster 2 (brief WPV reporting form); Yellow: Cluster 3 (detailed WPV reporting form)

M: Multiple choice item, B: Binary item, O: Open-ended item, X: Unknown
