# Supplementary material for: How to Adequately Report Workplace Violence in Healthcare Setting: A Systematic Review With Hierarchical Cluster Analysis of Workplace Violence Reporting Forms
Source: J Nurs Manag. 2026 Jun 28;2026:4803748. doi: 10.1155/jonm/4803748 (PMC13310381; doi:10.1155/jonm/4803748)
Supplement: Supplementary file 4 — Supporting Information 4 Supporting File 4: Agglomeration schedule. [file JONM-2026-4803748-s001.docx]

| **Stage** | **Cluster Combined** | | **Coefficient** | **Stage Cluster First Appears** | | **Next Stage** |
| --- | --- | --- | --- | --- | --- | --- |
|  | **Cluster 1** | **Cluster 2** |  | **Cluster 1** | **Cluster 2** |  |
| 1 | 17 | 22 | 0.525 | 0 | 0 | 19 |
| 2 | 12 | 18 | 0.381 | 0 | 0 | 9 |
| 3 | 10 | 13 | 0.370 | 0 | 0 | 7 |
| 4 | 9 | 21 | 0.333 | 0 | 0 | 18 |
| 5 | 5 | 7 | 0.333 | 0 | 0 | 6 |
| 6 | 5 | 11 | 0.286 | 5 | 0 | 12 |
| 7 | 8 | 10 | 0.275 | 0 | 3 | 11 |
| 8 | 15 | 20 | 0.275 | 0 | 0 | 13 |
| 9 | 12 | 14 | 0.272 | 2 | 0 | 12 |
| 10 | 1 | 2 | 0.243 | 0 | 0 | 13 |
| 11 | 6 | 8 | 0.228 | 0 | 4 | 16 |
| 12 | 5 | 12 | 0.210 | 6 | 9 | 15 |
| 13 | 1 | 15 | 0.203 | 10 | 8 | 17 |
| 14 | 16 | 19 | 0.190 | 0 | 0 | 16 |
| 15 | 4 | 5 | 0.172 | 0 | 12 | 17 |
| 16 | 6 | 16 | 0.169 | 11 | 14 | 18 |
| 17 | 1 | 4 | 0.159 | 13 | 15 | 19 |
| 18 | 6 | 9 | 0.157 | 16 | 4 | 20 |
| 19 | 1 | 17 | 0.151 | 17 | 1 | 20 |
| 20 | 1 | 6 | 0.112 | 19 | 18 | 21 |
| 21 | 1 | 3 | 0.001 | 20 | 0 | 0 |
